# Periodontitis may induce gut microbiota dysbiosis via salivary microbiota

**DOI:** 10.1038/s41368-022-00183-3

**Published:** 2022-06-23

**Authors:** Jun Bao, Lili Li, Yangheng Zhang, Min Wang, Faming Chen, Shaohua Ge, Bin Chen, Fuhua Yan

**Affiliations:** 1 Department of Periodontology, Nanjing Stomatological Hospital, Medical School of Nanjing University, No.30 Zhongyang Road, Nanjing, China; 2 State Key Laboratory of Military Stomatology & National Clinical Research Center for Oral Diseases & Shaanxi Clinical Research Center for Oral Diseases, Department of Periodontology, School of Stomatology, Fourth Military Medical University, Xi’an, China; 3 Department of Periodontology, School and Hospital of Stomatology, Cheeloo College of Medicine, Shandong University & Shandong Key Laboratory of Oral Tissue Regeneration & Shandong Engineering Laboratory for Dental Materials and Oral Tissue Regeneration, Jinan, China

**Keywords:** Periodontitis, Bacterial genomics, Bacterial infection

## Abstract

The aim of this study was to identify whether periodontitis induces gut microbiota dysbiosis via invasion by salivary microbes. First, faecal and salivary samples were collected from periodontally healthy participants (PH group, *n* = 16) and patients with severe periodontitis (SP group, *n* = 21) and analysed by 16S ribosomal RNA sequencing. Significant differences were observed in both the faecal and salivary microbiota between the PH and SP groups. Notably, more saliva-sourced microbes were observed in the faecal samples of the SP group. Then, the remaining salivary microbes were transplanted into C57BL6/J mice (the C-PH group and the C-SP group), and it was found that the composition of the gut microbiota of the C-SP group was significantly different from that of the C-PH group, with *Porphyromonadaceae* and *Fusobacterium* being significantly enriched in the C-SP group. In the colon, the C-SP group showed significantly reduced crypt depth and zonula occludens-1 expression. The mRNA expression levels of pro-inflammatory cytokines, chemokines and tight junction proteins were significantly higher in the C-SP group. To further investigate whether salivary bacteria could persist in the intestine, the salivary microbiota was stained with carboxyfluorescein diacetate succinimidyl ester and transplanted into mice. We found that salivary microbes from both the PH group and the SP group could persist in the gut for at least 24 h. Thus, our data demonstrate that periodontitis may induce gut microbiota dysbiosis through the influx of salivary microbes.

## Introduction

Periodontitis is an inflammatory disease with a high global prevalence.^1^ It is reported to be closely associated with many systemic diseases.^2^ Our previous research showed that the gut microbiota may mediate the impact of periodontitis on systemic diseases.^3–5^ However, the mechanism by which periodontal disease affects the intestinal microbiota needs to be further studied.

Previously, studies reported that periodontitis may influence systemic disease through blood circulation.^6,7^ The subgingival microbiota and its components enter circulation through the ulcerous periodontal pocket, which leads to low-grade systemic inflammation and impacts the development of systemic diseases/conditions, including the composition of the gut microbiota. Interestingly, we found that swallowing the periodontitis salivary microbiota could be the other pathway linking periodontitis and systemic disease.^5^ It is known that people swallow 1–1.5 L of saliva per day.^8^ In the case of patients with periodontitis, more pathogenic microbes may enter the gut along with saliva. Therefore, the swallowed pathogenic oral microbes may have a chance to disrupt the balance of the gut microbiota.

Recently, we reported that periodontitis salivary microbiota could worsen colitis in a DSS-induced mouse model of colitis.^5^ However, it is unknown whether the swallowed periodontitis salivary microbiota could disrupt the balance of the gut microbiota and disturb the homoeostasis of the immune response in the intestine; which is crucial for determining whether the salivary pathway is an independent pathway via which periodontitis affects the gut microbiota. Therefore, we transplanted periodontitis salivary microbiota into healthy mice to investigate this problem. First, we collected salivary and faecal samples from patients and analysed the correlation between the salivary microbiota and gut microbiota through a clinical study. Then, the human salivary microbes were given to healthy mice with a dose simulating the clinical situation. Through the above experimental design, we demonstrated for the first time that periodontitis can affect the gut microbiota and intestinal health of healthy animals through the salivary pathway. Finally, to further study the mechanism by which salivary microbes disturb the gut microbiota, we tested whether the salivary microbiota could persist in the intestine. We found that some salivary microbes could survive in the gut for at least 24 h.

This study provides new evidence for the mechanism by which periodontitis may affect systemic diseases and verifies for the first time that periodontitis salivary microbes could disrupt the balance of the healthy gut microbiota and disturb the homoeostasis of the immune response in the intestine. It also provides a new direction for research focusing on the impact of periodontitis on general health. In addition, it helps to uncover potential intervention targets and promotes the use of accurate intervention strategies.

## Results

### Clinical characteristics

**Fig. 1:**
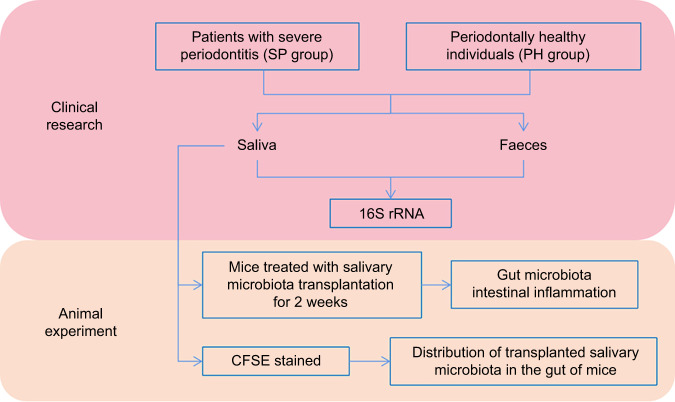
The experimental flowchart

The experimental flowchart is shown in Fig. 1. The inclusion and exclusion criteria are provided in Appendix Table 1. A total of 37 systemically healthy participants, namely, patients with severe periodontitis (SP group, *n* = 21) and periodontally healthy individuals (PH group, *n* = 16), were enrolled. The average age of those in the PH group (31.06 ± 8.13) was younger than that of those in the SP group (44.62 ± 10.42). Gender, body mass index, and smoking habits were similar between the PH and SP groups, as shown in Appendix Table 2 (*P* > 0.05). The debris index, plaque index, calculus index and gingival index in the SP group were significantly higher than those in the PH group, as shown in Appendix Table 3 (*P* < 0.05).

### Composition of the gut microbiota was altered in patients with periodontitis

**Fig. 2:**
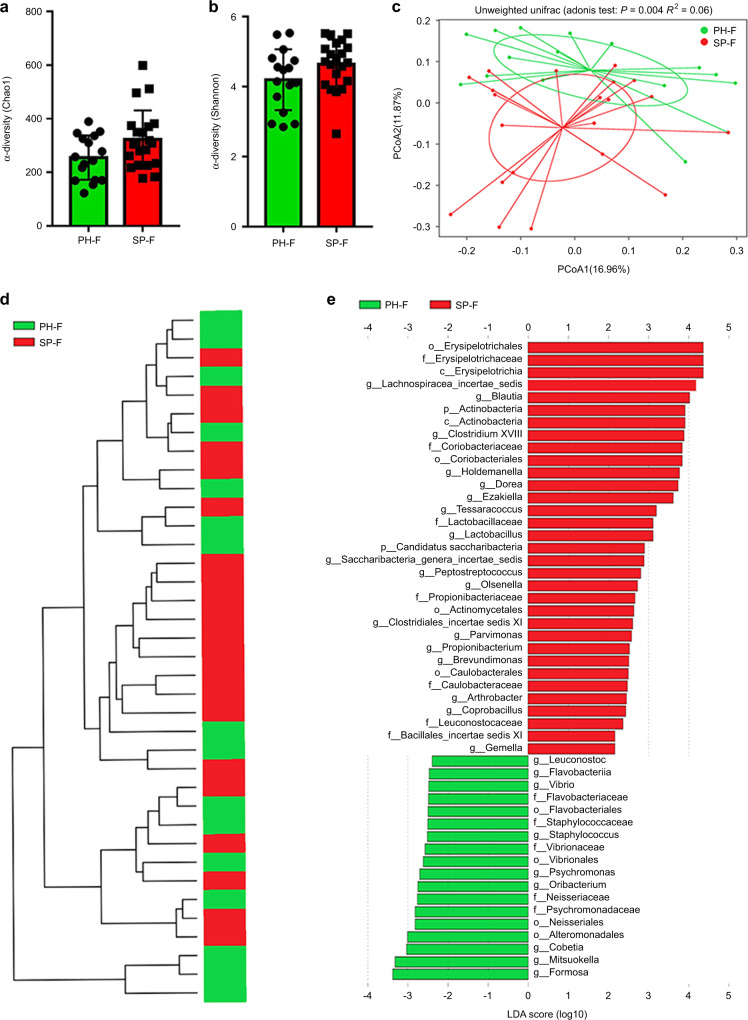
Gut microbiota alterations in the patients with periodontitis. **a**, **b** α diversity-Chao1 and Shannon indexes. Data were expressed as the mean ± SD. Statistical significance was determined through the Wilcoxon test. **c**, **d** Principal coordinate analysis (PCoA) and clustering analysis showed separate clustering (Adonis analysis, *P* < 0.05). **e** The SP group-enriched taxa are shown by a positive LDA score (red), while the taxa enriched in the PH group have a negative score (green). Taxa with an LDA score >2.0 are shown. *n* = 16 in the PH group and *n* = 21 in the SP group. *P < 0.05

To confirm the effect of periodontitis on the gut microbiota, the disparity in the faecal microbiota between the faeces from the PH group (PH-F, *n* = 16) and those from the SP group (SP-F, *n* = 21) was analysed by 16 S rRNA. The α-diversity indexes (Chao1 and Shannon) of the faecal microbiota indicated no significant difference between the PH and SP groups (*P* > 0.05) (Fig. 2a, b). Furthermore, a principal coordinate analysis (PCoA) plot and clustering tree revealed significant differences in the microbial composition between the two groups (*P* < 0.05) (Fig. 2c, d). According to linear discriminant analysis (LDA), *Erysipelotrichaceae*, *Lachnospiracea*_*incertae*_*sedis* and *Blautia* were enriched in the SP-F group, while *Formosa* and *Mitsuokella* were enriched in the PH-F group (*P* < 0.05) (Fig. 2e). These results showed that there were differences in the composition of the gut microbiota between the patients with periodontitis and periodontally healthy individuals.

### The periodontitis salivary microbiota with increased periopathogenic bacteria may enter the gut

**Fig. 3:**
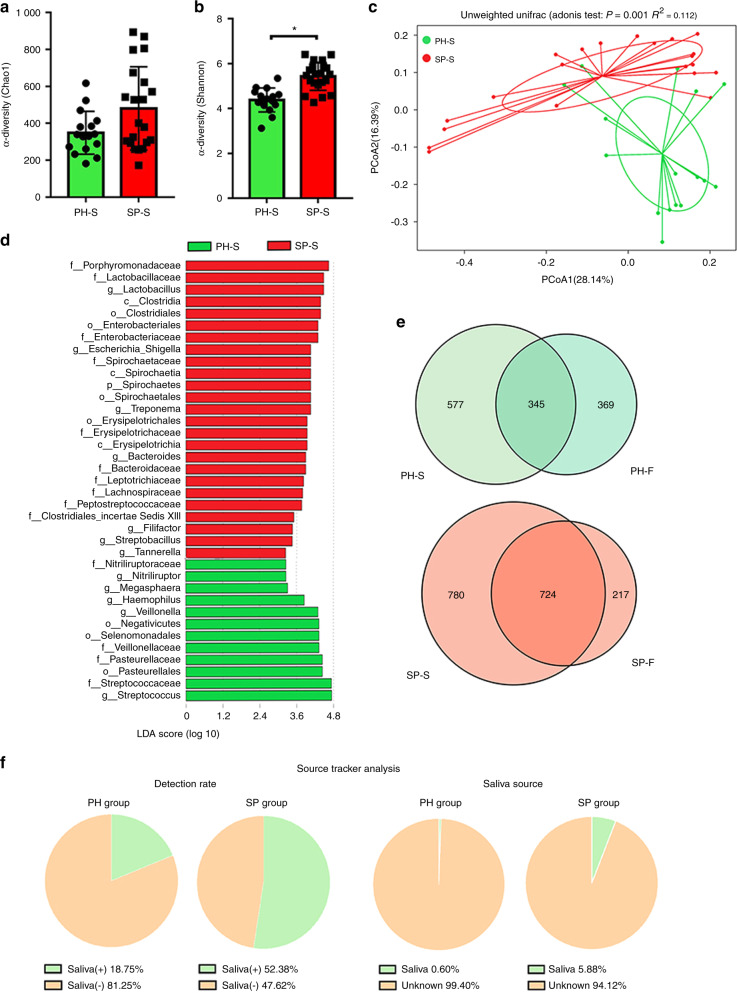
Periodontitis salivary microbiota with increased periopathogenic bacteria may enter the gut. **a**, **b** α diversity-Chao1 and Shannon indexes of the salivary microbiota. Statistical significance was determined using the Wilcoxon test. Data were expressed as the mean ± SD. **c** Principal coordinate analysis based on unweighted UniFrac distances (Adonis analysis, *P* < 0.05). **d** The LDA graph shows the significantly enriched bacteria in the PH-S (green bar) and SP-S (red bar) groups. Taxa with an LDA score >3.25 are shown. **e** The Venn diagram shows the shared bacteria of saliva and faeces. **f** The Source Tracker analysis shows saliva-derived microbiota in the faecal samples. “Saliva (+)” indicates the percentage of the PH and SP groups that had saliva-derived bacteria in their gut microbiota. “Saliva (−)” indicating the percentage of the PH and SP groups that did not have saliva-derived bacteria in their gut microbiota. “Saliva” refers to the percentage of saliva-derived bacteria in the faecal samples, and “unknown” refers to the percentage of other bacteria that were not derived from saliva. *n* = 16 in the PH group and *n* = 21 in the SP group. *P < 0.05

To confirm the previously mentioned hypothesis that periodontitis-induced changes in the gut microbiota may be due to swallowing saliva, the differences in the salivary microbiota of the PH (PH-S, *n* = 16) and SP (SP-S, *n* = 21) groups were compared by 16 S rRNA, and then Source Tracker analysis was performed to explore the link between the oral and gut microbiota. The Shannon index of the salivary microbiota was significantly increased in the SP group compared with the PH group (*P* < 0.05), whereas the Chao1 index of the faecal microbiota showed no significant difference between the PH and SP groups (*P* > 0.05) (Fig. 3a, b). PCoA indicated that the PH-S and SP-S samples clustered separately, as shown in Fig. 3c. *Porphyromonadaceae*, *Tannerella*, and *Treponema* were enriched in the SP group, and *Streptococcaceae*, *Veillonellaceae*, and *Pasteurellaceae* were enriched in the PH group according to LDA (*P* < 0.05) (Fig. 3d). In addition, a Venn diagram and Source Tracker analysis were performed to detect the common microbes in both the salivary and faecal samples (Fig. 3e, f). The number of shared operational taxonomic units (OTUs) between the salivary and faecal samples of the SP group was greater than that of the PH group, suggesting that the salivary and faecal microbiota of the patients with periodontitis were more similar (Fig. 3e). These results indicated that more oral-derived bacteria may colonise the gut in patients with periodontitis than in periodontally healthy individuals. Source Tracker analysis showed that salivary-derived bacteria were found in the gut microbiota in 18.75% of the periodontally healthy individuals and 52.38% of the patients with periodontitis, which indicates a higher detection rate in the SP group. Saliva-derived bacteria from periodontally healthy people accounted for 0.6% of the gut microbiota, while the proportion increased to 5.88% in the patients with periodontitis, suggesting that more salivary bacteria were retained in the gastrointestinal tract of the patients with periodontitis (Fig. 3f). The above results supported our hypothesis that periodontitis salivary microbes may induce gut microbiota dysbiosis by entering the gastrointestinal tract.

### Transplantation of periopathogenic microbiota from the SP patients altered the caecal microbiota and induced low-grade intestinal inflammation in mice

**Fig. 4:**
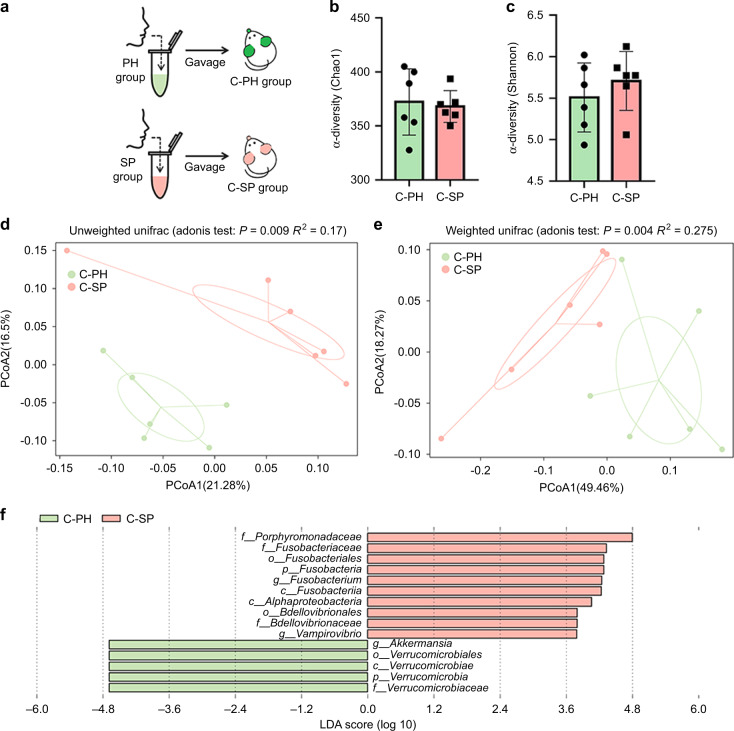
Salivary microbiota from the patients with periodontitis led to gut microbiota dysbiosis in mice. **a** The experimental flowchart in the C-PH (*n* = 6) and C-SP (*n* = 6) groups. **b**, **c** α diversity-Chao1 and Shannon indexes of the caecal microbiota. Data were expressed as the mean ± SD. Statistical significance was determined using the Wilcoxon test. **d**, **e** The principal coordinate analysis was based on the unweighted UniFrac distance (Adonis analysis, *P* < 0.05). **f** The LDA graph shows the taxonomic differences in the C-PH and C-SP groups. Taxa with an LDA score >2.0 are shown

To further investigate the effects of periodontitis-associated salivary microbes on the gut microbiota and mucosal barrier, the salivary microbiota from the PH and SP groups were transplanted into mice (C-PH group, *n* = 6; C-SP group, *n* = 6) through oral gavage, and the caecal microbiota and gut mucosal barrier were analysed two weeks later (Fig. 4a). The gut microbiota was altered significantly in the C-SP group. The Chao1 and Shannon indexes of the caecal microbiota showed no significant differences between the two groups (*P* > 0.05) (Fig. 4b, c). The caecal microbiota of the two groups were significantly different according to PCoA (*P* < 0.05) (Fig. 4d, e). *Akkermansia* was enriched in the C-PH group, while *Porphyromonadaceae* and *Fusobacterium* were enriched in the C-SP group according to LDA (*P* < 0.05) (Fig. 4f).

**Fig. 5:**
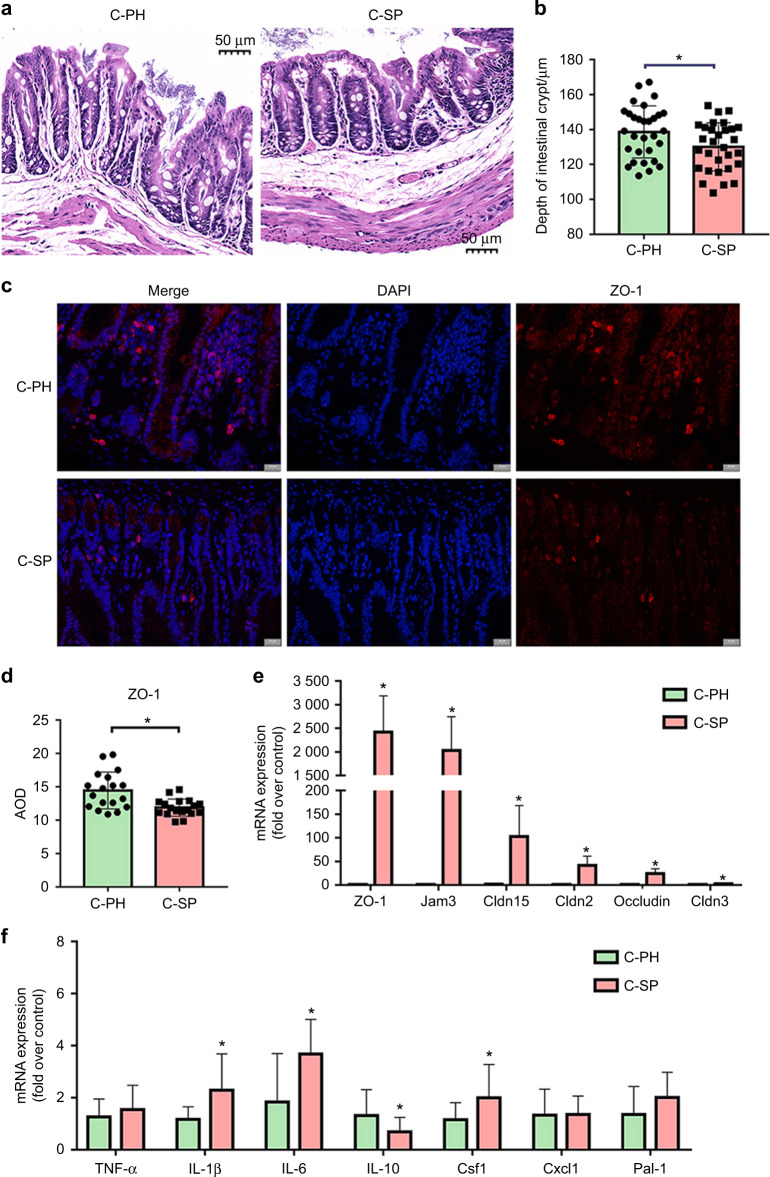
Salivary microbiota from the patients with periodontitis affected the gut barrier of mice. **a** Representative images of the HE-stained colon sections. **b** Measurements of the colonic crypt depth of the HE-stained colon sections. Scale bar, 50 µm. **c**, **d** Representative images and measurements of immunofluorescence-stained sections of proximal colon tissue targeting ZO-1 (red) and DAPI (blue). Scale bar, 20 µm. **e**, **f** The fold changes in the mRNA expression of pro-inflammatory factors, chemokines, and tight junction proteins in proximal colon tissue. Data were expressed as the mean ± SD (*n* = 4–6 per group). Statistical significance was determined using the *t*-test. *P < 0.05

Moreover, changes in the gut mucosal barrier were observed in the C-SP group. The crypt depth of the gut mucosal barrier was significantly reduced in the C-SP group compared with the C-PH group (*P* < 0.05) (Fig. 5a, b). Immunofluorescence staining showed that the expression levels of zonula occludens-1 (ZO-1) decreased significantly in the C-SP group (Fig. 5c, d). The mRNA expression levels of tight junction proteins, such as junctional adhesion molecule 3 (Jam3), ZO-1, claudin-2 (Cldn2), claudin-3 (Cldn3), claudin-15 (Cldn15) and occludin, were significantly increased in the C-SP group compared with the C-PH group (*P* < 0.05) (Fig. 5e). In addition, the mRNA expression levels of pro-inflammatory factors and chemokines, such as interleukin-1β (IL-1β), interleukin-6 (IL-6) and colony-stimulating factor 1 (Csf1), were significantly upregulated in the C-SP group compared with the C-PH group (*P* < 0.05) (Fig. 5f). Anti-inflammatory cytokines such as interleukin-10 (IL-10) showed a significant decrease in the C-SP group compared with the C-PH group (*P* < 0.05) (Fig. 5f). There were no significant differences in the expression levels of (c-x-c motif) ligand 1 (Cxcl1) and plasminogen activator inhibitor type-1 (PAI-1) between the two groups (*P* > 0.05) (Fig. 5f). The above results indicated that periodontitis-induced gut microbiota dysbiosis and intestinal low-grade inflammation through the salivary microbiota.

### The periodontitis salivary microbiota could remain in the caecum

**Fig. 6:**
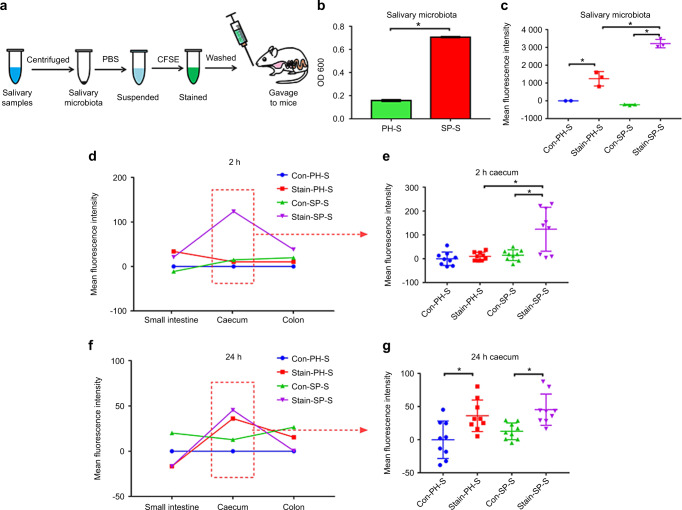
The periodontitis salivary microbiota could persist in the intestinal tract for at least 24 h. **a** An experimental flowchart indicating how the salivary microbiota is processed and transplanted into mice (*n* = 6 in each group). **b** The concentrations of microbial suspensions from the PH and SP group were measured by OD_600_. Data were expressed as the mean ± SD. Statistical significance was determined using the *t*-test. **c** The mean fluorescent intensity of unstained and stained microbial suspensions from the PH and SP groups. Data were expressed as the mean ± SD and were compared to data from the unstained PH salivary microbiota. Statistical significance was determined using one-way ANOVA. **d**–**g** The distribution of fluorescence-positive bacteria in the gastrointestinal tract (small intestine, caecum, and colon) at 2 and 24 h after gavage. Data were compared to the Con-PH-S group and expressed as the mean or mean ± SD. Statistical significance was determined using one-way ANOVA. *P < 0.05

Since determining whether salivary microbes can survive in the intestine will facilitate our understanding of how the periodontitis salivary microbiota affected the gut microbiota, we stained the salivary bacteria with a live bacteria-specific dye: carboxyfluorescein diacetate succinimidyl ester (CFSE) and traced the fluorescent bacteria in the intestine of mice after gavage (*n* = 6 for each group), as shown in Fig. 6a. The concentration of the microbial suspension in the SP group (0.707 × 10^9^ CFU·mL^−1^) was significantly higher than that in the PH group (0.158 × 10^9^ CFU per mL) (Fig. 6b). The mean fluorescence intensity of the stained microbial suspensions (2217.43 ± 405.05 in the Stain-PH-S group, 4195.47 ± 236.66 in the Stain-SP-S group) were significantly higher than those of the unstained microbial suspensions (975.66 ± 1.16 in the Con-PH-S group, 752.67 ± 31.19 in the Con-SP-S group) after CFSE staining (Fig. 6c).

After 2 h, the mean fluorescence intensity of the caecal content was significantly higher in the Stain-SP-S group than in the Con-SP-S and Stain-PH-S groups (*P* < 0.05) (Fig. 6d, e), indicating that more saliva-derived bacteria from the SP group entered the caecum within 2 h. After 24 h, the mean fluorescence intensity of the caecal microbiota was significantly higher in the stained group than in the unstained control group (*P* < 0.05) (Fig. 6f, g). These data indicated that salivary bacteria were retained in the gastrointestinal tract for at least 24 h.

## Discussion

Periodontitis has a high prevalence and endangers the entire body.^9–11^ In the present clinical study, we found that periodontitis may induce gut microbiota dysbiosis and intestinal inflammation by the translocation of salivary microbes. The results of the present study further supported the importance of a healthy periodontal status. Furthermore, the mechanism underlying the translocation of salivary microbes to the gut serves as the foundation for optimising the understanding of how periodontitis affects the gut microbiota. Profoundly, the treatment of periodontitis may provide a new potential method for treating refractory gut microbiota-associated systemic diseases.

The present study suggested that periodontitis causes changes in the gut microbiota. To a large extent, this finding was consistent with a clinical study.^12^ Notably, the β-diversity revealed significant differences in the faecal microbial composition between the PH and SP groups in the present study (Fig. 2c). However, no significant difference was reported between individuals presenting chronic periodontitis (*n* = 23), gingivitis (*n* = 14) and periodontal health (*n* = 7) in the study.^12^ We speculate that this discrepancy may be due to the different inclusion criteria for periodontitis individuals. The former study included both moderate and severe periodontitis, resulting in greater intra-group differences and making it hard to draw a conclusion with small sample sizes. In this study, we included only patients with severe periodontitis, which could minimise the intra-group differences, providing a more significant systemic effect. Hence, multi-centre and large-sample clinical studies are needed to further clarify these specific differences in the future. The above evidence also suggests that the severity of periodontitis may be closely related to the changes in the intestinal microbiota caused by periodontitis. Therefore, it is necessary to design relevant experiments to confirm this hypothesis in the future.

As previously described, the present findings suggested that differences exist in the gut microbiota between the PH and SP groups. Certainly, studying the mechanism by which periodontitis affects the gut microbiota is the fundamental strategy for revealing the differences in the gut microbiota between the two groups. Considering that adults swallow ~10^12^ bacteria per day^13,14^ and that saliva-derived microorganisms may colonise the gut, the important mechanism underlying changes in the gut microbiota of patients with periodontitis may be related to the entry of periodontal bacteria into the intestine via saliva. Therefore, the salivary microbiota of the PH and SP groups were compared. Significantly higher numbers of anaerobic bacteria, including *Porphyromonadaceae*, *Tannerella* and *Treponema*, were enriched in the SP-S group (Fig. 3d), which was in accordance with the typical characteristics of periodontopathic microbiota in subgingival dental plaque.^15,16^ These findings were consistent with those of previous studies.^5,17–20^ Source Tracker analysis revealed that more oral-derived bacteria existed in the altered faecal samples of the SP group (Fig. 3f), and Iima et al. also revealed the similarity of the oral and gut microbiota,^21^ supporting our hypothesis that the salivary microbiota of patients with periodontitis may induce gut microbiota dysbiosis by entering the gastrointestinal tract.

To verify the aforementioned hypothesis, the salivary microbiota of the PH and SP groups was transplanted into mice via gavage. Similar to the results of human studies, there were significant differences in the gut microbiota of the mice (Fig. 4d, e). The abundance of *Porphyromonadaceae* and *Fusobacterium*, which are co-pathogens in periodontitis and intestinal inflammation,^22–24^ was significantly enriched in the gut of the C-SP group (Fig. 4f). Interestingly, *Porphyromonadaceae* was also enriched in the salivary samples from the humans, revealing the close relationship between the salivary and gut microbiota. Additionally, *Fusobacterium* possesses adhesion and aggregation functions and can bind to most oral bacteria.^25^ *Fusobacterium* can also adhere to some gut pathogenic bacteria,^26^ which may disturb the gut microbiota, lead to intestinal inflammation,^27,28^ and potentially serve as one of the biomarkers for detecting early intestinal bowel disease.^27,29,30^ These results indicated that saliva-derived putative periodontopathic microbiota may enter the gut.

To study the effect of the salivary microbiota on the intestine, changes in intestinal inflammation levels and permeability were evaluated. The mRNA expression levels of cytokines and chemokines increased in the C-SP group (Fig. 5f), suggesting that the gut was in a state of low-grade inflammation; in addition, the intestinal mucosal barrier was damaged, the expression levels of ZO-1 were decreased according to IF staining, and the crypt depth was decreased in the C-SP group (Fig. 5a–d), which was consistent with other studies.^31–34^ However, the mRNA expression levels of tight junction proteins, as important markers for detecting the permeability of the intestinal mucosal barrier, were elevated in the C-SP group (Fig. 5e). This seemed contradictory, but it may actually be a compensatory response to low-grade intestinal inflammation.^35^ When the intestinal barrier is challenged by an external stimulus, the tight junction structure of the intestinal epithelium can be disrupted, and the expression of tight junction proteins decreases.^36^ In addition, intestinal epithelial cells upregulate the gene expression level of those tight junction proteins in an attempt to compensate for the destruction of the tight junction. Thus, a paradoxical situation emerges: the protein levels of tight junction proteins may be decreased, while the gene expression levels may be increased, which was reported in a previous study.^37^ This could be one stage of the destruction of intestinal epithelial cells. When the function of epithelial cells is further damaged and exceeds the compensatory ability, both the tight junction protein levels and the gene expression levels will be reduced.

The aforementioned results further verified that the periodontitis salivary microbiota can affect the homoeostasis of the gut microbiota by transplantation. However, the underlying mechanism by which the salivary microbiota affects the gut microbiota is unclear. Thus, it was critical to explore whether salivary bacteria could persist in the intestine. The salivary microbiota was stained with CFSE to trace the bacterial distribution in the intestine. CFSE is a live bacteria-specific dye that can easily pass through the membrane and transform into a fluorescent substance in living bacteria but not in dead bacteria. The fluorescent product is stable and can be retained within live bacteria to trace salivary bacteria.^38^ We found that salivary bacteria could be detected in the intestinal tract of mice for at least 24 h (Fig. 6 and Appendix Fig. 1), which suggested that periodontal bacteria can enter the intestinal tract through saliva and may survive.

There may be two main mechanisms by which periodontitis affects the intestine: the host immune response or ectopic colonisation of oral bacteria.^6,34^ Previous studies have focused mainly on the link between the periodontal pocket and blood circulation.^6,7^ Subgingival microbes may invade the circulation through the ulcerous periodontal pocket, leading to low-grade systemic inflammation, thereby resulting in intestinal immune imbalance and alteration of the gut microbiota.^39^ In contrast, some researchers have argued that putative periodontal pathogens may enter the intestine along with saliva, further disturbing the homoeostasis of the gut microbiota.^13,34,40^

The saliva-gut pathway could be another pathway through which periodontitis affects the gut microbiota. However, it is unclear whether the saliva-gut pathway is an independent mechanism unrelated to the periodontal pocket-blood circulation pathway. Although preliminary tests to examine this theory by orally inoculating *Porphyromonas gingivalis*^41^ and *Actinobacillus actinomycetemcomitans*^42^ have been reported, the use of oral inoculation (especially using 2% carboxymethyl-treated cells to promote bacterial colonisation) may not be able to rule out possible mechanisms in which the periodontal pocket-blood circulation pathway is involved. In addition, it will be very difficult to simulate the actual state of these patients with a single bacterial species. Instead, we tried to simulate the clinical situation by isolating the salivary microbiota from patients with periodontitis and then transplanted the microbial suspension via gavage into mice with a healthy periodontium rather than the use of a ligation model. Hence, we may have effectively avoided the influence of systemic immune-inflammatory reactions irritated by local periodontal inflammation on the intestine; in addition, a combination of salivary microbes is closer to clinical conditions than that of a single strain. Thus, we were able to successfully observe that the swallowed oral flora could independently cause an imbalance in the gut microbiota and disturb the intestinal mucosal barrier as well. We have demonstrated the independence of the saliva-gut pathway on the impact of periodontitis on the gut microbiota.

Since it is difficult to collect saliva samples from mice with periodontitis, human saliva was alternatively used for the experiment. This method has limitations: the oral microbiota and gut microbiota of humans differ from those of mice.^43^ Thus, human oral microbiota-associated, germ-free and faecal microbiota transplantation models may be considered to explore the relationship between the oral and gut microbiota in future research.^3,40,43,44^

In conclusion, the present study showed that periodontitis may induce gut microbiota dysbiosis through the influx of salivary microbes and that the microbes in saliva that enter and colonise the intestine may be an important reason for this phenomenon. Further research should focus on which bacteria are involved and how they affect this process.

## Materials and Methods

### Study population and sample collection

The participants in this study were recruited from Nanjing Stomatological Hospital, Medical School of Nanjing University, and the study was approved by the Ethical Committee of Nanjing Stomatological Hospital, Medical School of Nanjing University (2019NL-008(KS)). There were 21 patients with periodontitis in the SP group and 16 periodontally healthy individuals in the PH group.

The inclusion and exclusion criteria are presented in Appendix Table 1.^12^ At the first visit, the participants received a professional clinical examination and a comprehensive treatment plan. The debris index (DI), plaque index (PLI), calculus index (CI), and gingival index (GI) were assessed.^5^ The participants were individually informed about the nature of the study and its risks and benefits, and each participant completed a questionnaire and signed an informed consent form. They were informed not to drink or eat anything, not to brush their teeth or gargle within 8 h after brushing their teeth the night before the second visit. Salivary and faecal samples were collected during the second visit. At this visit, the unstimulated saliva of each donor was collected by drooling in a 50-mL sterile centrifuge tube every 2 min until the volume of saliva was more than 4 mL within 15 min, and this step was repeated twice. The appearance of saliva was obviously different (Appendix Fig. 2a). Since it was difficult to ensure the collection of fresh saliva in time for experiments due to the uncontrollability of sample collection, we used a conventional method for saliva preservation based on relevant literature.^45^ Accordingly, 4 mL of each saliva sample was mixed with an equal volume (w/v) of phosphate-buffered saline (PBS) containing 20% glycerol/PBS and stored at −80 °C. To further determine whether the salivary bacteria were still viable after freezing, the saliva from both the PH group and SP group were cultured (Appendix Fig. 2a– c). The methods and materials for the assessment of salivary bacterial counts are described in detail in the appendix. The results demonstrate that there was no significant reduction in the CFUs of salivary bacteria in both the PH group and SP group (Appendix Fig. 2d, e). The first fresh mid-piece of faecal sample, about the size of a soybean, was collected with a faecal collector. Faecal and individual saliva samples were frozen using dry ice and stored at −80 °C for 16 S ribosomal RNA (16 S rRNA) analysis. Both groups received oral hygiene instructions after sample collection.

### Isolation of the salivary microbiota

Since both patients with periodontitis and periodontally healthy individuals swallow 1–1.5 L of saliva per day, we calculated the volume of saliva given to mice according to the weight ratio of humans and mice to simulate the clinical situation. More specifically, adults secrete 1–1.5 L of saliva every day, and the weight of an adult is ~60 kg. The weight of a mouse is ~20 g. Therefore, each mouse received 0.5 mL (1 500 mL/60 000 g × 20 g) of pooled saliva per day via gavage.

On the day of oral gavage, the frozen saliva samples were thawed. An equal volume of 8 mL of the saliva samples (4 mL of saliva and 4 mL of glycerol/PBS) from the participants in each group were pooled, well mixed, divided into 1 mL (0.5 mL of saliva and 0.5 mL of glycerol/PBS) per tube, and subsequently used for the animal experiments. Then, the divided saliva samples were centrifuged at 1 000 × *g* for 5 min to remove food residues and exfoliated cells and then centrifuged at 3 300 × *g* for 10 min at 4 °C to collect the microbiota. Then, the salivary microbiota was suspended in PBS to give an oral gavage of 200 µL per mouse, which is a proper volume for mice.^40^

### CFSE staining of the salivary microbiota

The concentration of the microbial suspension was measured using the OD_600_ (OD_600_ = 1.0 approximately corresponding to 10^9^ CFU/mL).^46^ Microbial suspensions of the PH and SP groups were stained with 5-(and 6)-CFSE (Dojindo Laboratories, Japan) according to the manufacturer’s instructions. Briefly, the concentration of the microbial suspension was adjusted to 10^7^ CFU·mL^−1^. Then, CFSE was added to a final concentration of 5 μmol·L^−1^. The suspension was mixed thoroughly and incubated for 30 min at 37 °C. Labelling was stopped by adding PBS and washing the bacteria three times to remove excess fluorescence signal. The CFSE-stained bacteria were centrifuged and collected. Then, the bacteria were resuspended in PBS prior to oral gavage.

The mean fluorescence intensity of unstained and stained microbial suspensions was detected by using a microplate reader (Thermo Fisher Scientific, Waltham, USA) with excitation at 496 nm and emission at 516 nm. The unstained PH salivary microbiota was treated as a negative control.

### Animal experimental design

Six-week-old male C57BL/6 J wild-type mice (Changzhou Cavens Lab Animal Company, Changzhou, China) were raised with a standard diet and lifestyle in a specific pathogen-free environment in the Experimental Animal Center of Nanjing Agricultural University. This study followed the Animal Research: Reporting of In Vivo Experiments (ARRIVE) guidelines. A 1-week adaptation period was allowed. The protocol for animal experiments was approved by the Ethical Committee of Nanjing Stomatological Hospital, Medical School of Nanjing University (PZW2020010).

Twelve mice were randomised into two groups: the C-PH and C-SP groups. Then, microbial suspensions from the PH and SP groups were transplanted into the mice via oral gavage of 200 µL per mouse, once a day, for 2 weeks. The mice were euthanized, and samples were collected 2 weeks later.

For mechanism exploration, twenty-four mice were randomised into four groups: Stain-PH-S group, Stain-SP-S group, Con-PH-S group, and Con-SP-S group. A 200-µL aliquot of microbial suspension stained with CFSE was orally gavaged once to the mice in the Stain-PH-S group and Stain-SP-S group via oral gavage. The same volume of the unstained salivary microbial suspension was transplanted into the Con-PH-S and Con-SP-S groups via oral gavage. Half of the mice from each group were euthanized after 2 h. The remaining mice were euthanized 24 h later. The fresh contents of the intestine, caecum and colon were collected.

### 16S rRNA gene analysis

Total DNA from saliva samples of the participants in the PH and SP groups was isolated using an E.Z.N.A.^®^ Soil DNA Kit (Omega Bio-Tek, Norcross, USA). DNA was extracted from each faecal and caecal sample using an improved protocol based on the QIAamp Fast DNA Stool Mini Kit (Qiagen, Germany). In detail, added 1 mL of InhibitEX Buffer to each 200 mg of faecal sample. The mixture was homogenised and vortexed (60 Hz) twice for 1 min using a Homogeneous instrument (FASTPREP-24, Aosheng Biotech, China). The sample was centrifuged for 1 min to pellet stool particles. Then, 600 μL of the supernatant was pipetted into a new 2 mL microcentrifuge tube containing 25 µL Proteinase K. Finally, the DNA purification was performed according to the manufacturer’s instructions. The specific V4-V5 region of the 16 S rRNA gene in the human samples was amplified with primers 515 F (5-GTGCCAGCMGCCGCGGTAA-3) and 907 R (5-CCGTCAATTCMTTTRAGTTT-3), and the specific V3-V4 region of the 16 S rRNA gene in the animal samples was amplified with primers 341 F (5-CCTACGGGRSGCAGCAG-3) and 806 R (5-GGACTACVVGGGTATCTAATC-3) to construct 16 S rRNA gene libraries, which were then sequenced by using the Illumina PE250 platform to analyse the raw sequencing data. Negative controls consisting of sterile water were used for DNA extraction and amplification.

All the samples were successfully sequenced. Assembled tags and trimmed barcodes and primers were further checked. Sequences were filtered according to quality scores. Low-quality 16 S tags <220 bp or >500 bp, such that the average Phred score of bases was <20 (Q20) or >3 ambiguous N, were removed. After enumerating the copy number of tags and removing the repeated tags, only the sequences with frequencies greater than 1 were clustered into OTUs. OTUs were defined as sharing 97% sequence identity using UPARSE (http://drive5.com/uparse/), and chimeric sequences were analysed and removed by using Usearch (version 7.0.1090). Sequences were analysed by using the RDP Classifier (http://rdp.cme.msu.edu/) against the RDP database (http://rdp.cme.msu.edu/) with a confidence threshold of 80%. An OTU profiling table and alpha diversity analyses were performed mainly using QIIME (version 1.9.1).^4,47^ LDA was used to estimate the impact of the abundance of each bacterial taxa on the difference between two groups by using LDA effect size (LEfSe) tools.^48^ Source Tracker analysis was based on the Bayesian method to explore the source of the microbiome in the target sample as previously described.^49,50^ This process was performed by RealBio Technology Company, Shanghai, China.^4^

### Detection of CFSE-stained bacteria in gastrointestinal contents

The contents of the small intestine, caecum and colon from the Con-PH-S, Con-SP-S, Stain-PH-S and Stain-SP-S groups were weighed. Each gut sample was added to 1 mL of PBS. Then, the samples were homogenised. The particles were removed by 100 µm pore size sieves. The supernatants were centrifuged at 6 000 × *g* for 15 min to collect the gut microbiota as previously described.^51^ The microbiota was suspended in PBS (w/v: 0.1 g contents per 500 µL) and diluted 10-fold. A 200-µL aliquot of microbiota was added to each well in 96-well black plates. The mean fluorescence intensity of the gut contents in each group was detected by using a microplate reader (Thermo Fisher Scientific, Waltham, USA) with excitation at 496 nm and emission at 516 nm. The unstained Con-PH-S group was treated as a negative control.

### Histological analysis

Proximal colon tissues of the mice were fixed with 4% paraformaldehyde (Boster Biological Technology Co., Ltd, Wuhan, China). These tissues were then embedded in paraffin and sectioned (5 µm thick). HE staining images were captured by using a Pannoramic scanner (3DHistech Ltd., Budapest, Hungary). The colonic crypt depth was evaluated by Caseviewer (Version 2.2.0.85100, 3DHISTECH Ltd., Budapest, Hungary), and it was defined as the distance from the intestinal crypt mouth to the base. The depth of 5 well-oriented crypts was measured in each section as previously described.^52^

Immunofluorescence staining with rabbit anti-ZO-1 antibody (1:100, Proteintech, USA) and DAPI (Servicebio, China) was performed overnight at 4 °C. Then, the sections were washed with PBS and incubated with the corresponding secondary antibody (Cyanine 3-conjugated goat anti-rabbit IgG, Servicebio). Images were collected using a Nikon Eclipse TI-SR fluorescence microscope with an identical camera, microscope lens, and light settings according to a previously described method.^5^ Fluorescence intensity was analysed by ImageJ software (Version 1.51). In detail, images were inverted from RGB colour type to 8-bit format, and a region of interest was selected for each slice. Then, the optical density of each image was calibrated. The average optical density (AOD) was calculated (AOD = integrated optical density/area × 100) as previously described.^53^

### Real-time PCR

Total mRNA was extracted from proximal colon tissues using TRIzol reagent (Tiangen Biotech Co. Ltd., Beijing, China) and then reverse transcribed into cDNA using the PrimeScript^®^ RT Master Mix kit (Takara, Shiga, Japan). The primers used are presented in Appendix Table 4. Quantitative real-time PCR (qPCR) was performed on Viia 7 Quantitative Real-time PCR machines (Applied Biosystems, Foster City, USA) by using SYBR Green qPCR Mix (Bio-Rad, Hercules, USA). The signals were normalised against the housekeeping gene GAPDH. The relative expression of mRNA was calculated using the 2^−ΔΔCT^ method.^3,54^

### Statistical analysis

Data were expressed as the mean or mean ± standard deviation (SD). Participant demographics were analysed using Student’s *t*-test or the chi-square test. The Wilcoxon test was used to compare differences in α-diversity between relevant groups. Measurement data were assessed with the *t*-test or one-way ANOVA. All computations were performed using GraphPad Prism software (v 7.0, GraphPad Software, Inc., San Diego, CA): *P < 0.05.

## Supplementary information

Appendix

## Data Availability

The raw microbiome sequencing data were uploaded to the NCBI Sequence Read Archive database with accession no. PRJNA783440.
