# Supplementary material for: Periodontitis may induce gut microbiota dysbiosis via salivary microbiota
Source: Int J Oral Sci. 2022 Jun 23;14:32. doi: 10.1038/s41368-022-00183-3 (PMC9217941; doi:10.1038/s41368-022-00183-3)
Supplement: Supplementary file 1 — Appendix [file 41368_2022_183_MOESM1_ESM.docx]

**Appendix**

| **Appendix Table 1 Inclusion and exclusion criteria** |
| --- |
| **Inclusion criteria** |
| Periodontal healthy group: |
| 1) Han population aged 25-65; |
| 2) At least 18 natural teeth in the oral cavity; |
| 3) Periodontally healthy: clinical gingival health on an intact periodontium. |
| Severe periodontitis group: |
| 1) Han population aged 25-65; |
| 2) At least 18 natural teeth in the oral cavity; |
| 3) Generalized periodontitis: >30% of sites have bone resorption and attachment loss among all teeth; |
| 1. Severe periodontitis: Two or more affected teeth PD > 6 mm; CAL > 5 mm; RBL exceeds 1/2 of the length of the root. |
| **Exclusion criteria** |
| 1) Received periodontal treatment in the past 1 year; |
| 2) Received antibiotic treatment in the past 6 months; |
| 3) Received non-steroidal anti-inflammatory drugs within 3 months; |
| 4) Suffer from systemic diseases or conditions that affect the gut microbiota, such as chronic gastrointestinal diseases, diabetes, cardiovascular, cerebrovascular diseases, hypertension, nephritis, haematological diseases, immunodeficiency, and obesity (BMI ≥ 30); |
| 5) Suffer from other serious oral diseases; |
| 6) Heavy smoker (> 10 PCS/day); |
| 7) Pregnant or nursing; |
| 8) People with a special diet preference (vegan or pure meat eaters). |

| **Appendix Table 2 Participant demographics** | | | |
| --- | --- | --- | --- |
|  | **PH group** | **SP group** | **P-value** |
|  | **(n=16)** | **(n=21)** | **(PH vs SP)** |
| Age (mean years ± SD) | 31.06 ± 8.13 | 44.62 ± 10.42 | <0.05^A^ |
| Male, % (n) | 56.25 (9) | 66.67 (14) | >0.05^B^ |
| Female, % (n) | 43.75 (7) | 33.33 (7) |  |
| Body-mass index, mean ± SD | 22.97 ± 3.60 | 23.89 ± 3.23 | >0.05^A^ |
| Nonsmoker, % (n) | 93.75 (15) | 76.19 (16) | >0.05^B^ |
| Smoker, % (n) | 6.25 (1) | 19.05 (4) |  |
| Exsmoker, % (n) | 0 (0) | 4.76 (1) |  |

^A^t test. ^B^Chi-square test.

| **Appendix Table 3 The description of participants' oral status** | | | | | |
| --- | --- | --- | --- | --- | --- |
| **Number** | **Diagnosis** | **DI** | **PLI** | **CI** | **GI** |
| P1 | Periodontitis (severe, generalized) | 2 | 3 | 3 | 3 |
| P2 | Periodontitis (severe, generalized) | 1 | 1 | 2 | 2 |
| P3 | Periodontitis (severe, generalized) | 2 | 2 | 3 | 3 |
| P4 | Periodontitis (severe, generalized) | 2 | 3 | 3 | 3 |
| P5 | Periodontitis (severe, generalized) | 2 | 3 | 3 | 3 |
| P6 | Periodontitis (severe, generalized) | 2 | 2 | 2 | 2 |
| P7 | Periodontitis (severe, generalized) | 2 | 2 | 2 | 2 |
| P8 | Periodontitis (severe, generalized) | 1 | 1 | 2 | 2 |
| P9 | Periodontitis (severe, generalized) | 2 | 2 | 2 | 2 |
| P10 | Periodontitis (severe, generalized) | 1 | 1 | 2 | 2 |
| P11 | Periodontitis (severe, generalized) | 2 | 2 | 2 | 2 |
| P12 | Periodontitis (severe, generalized) | 2 | 2 | 3 | 3 |
| P13 | Periodontitis (severe, generalized) | 2 | 2 | 2 | 3 |
| P14 | Periodontitis (severe, generalized) | 2 | 2 | 2 | 2 |
| P15 | Periodontitis (severe, generalized) | 2 | 2 | 3 | 3 |
| P16 | Periodontitis (severe, generalized) | 2 | 2 | 2 | 2 |
| P17 | Periodontitis (severe, generalized) | 2 | 2 | 2 | 2 |
| P18 | Periodontitis (severe, generalized) | 2 | 2 | 3 | 3 |
| P19 | Periodontitis (severe, generalized) | 2 | 1 | 2 | 2 |
| P20 | Periodontitis (severe, generalized) | 2 | 2 | 2 | 2 |
| P21 | Periodontitis (severe, generalized) | 2 | 2 | 2 | 3 |
| H1 | Healthy | 0 | 0 | 0 | 0 |
| H2 | Healthy | 0 | 0 | 0 | 0 |
| H3 | Healthy | 1 | 1 | 0 | 0 |
| H4 | Healthy | 1 | 1 | 0 | 0 |
| H5 | Healthy | 0 | 0 | 0 | 0 |
| H6 | Healthy | 0 | 0 | 0 | 0 |
| H7 | Healthy | 0 | 0 | 0 | 0 |
| H8 | Healthy | 0 | 0 | 0 | 0 |
| H9 | Healthy | 0 | 0 | 0 | 0 |
| H10 | Healthy | 0 | 0 | 0 | 0 |
| H11 | Healthy | 0 | 0 | 0 | 0 |
| H12 | Healthy | 0 | 0 | 0 | 0 |
| H13 | Healthy | 1 | 1 | 0 | 0 |
| H14 | Healthy | 1 | 1 | 0 | 0 |
| H15 | Healthy | 0 | 0 | 0 | 0 |
| H16 | Healthy | 0 | 0 | 0 | 0 |
| *Abbreviations:* DI, Debris index; PLI, Plaque index; CI, Calculus index; GI, Gingival index. | | | | | |

| **Appendix Table 4 Primers for quantitative PCR** | | |
| --- | --- | --- |
| **Gene name** | **Forward primer sequence (5’-3’)** | **Reverse primer sequence (5’-3’)** |
| *Gapdh* | AGGTCGGTGTGAACGGATTTG | TGTAGACCATGTAGTTGAGGTCA |
| *TNF-α* | CATGAGCACAGAAAGCATGATCCG | AGCAGGAATGAGAAGAGGCTGAG |
| *IL-1β* | CCCAACTGGTACATCAGCAC | TCTGCTCATTCACGAAAAGG |
| *IL-6* | AGTTGCCTTCTTGGGACTGA | TCCACGATTTCCCAGAGAAC |
| *IL-10* | ACTCTTCACCTGCTCCACTG | GCTATGCTGCCTGCTCTTAC |
| *Csf1* | GTGTCAGAACACTGTAGCCAC | TCAAAGGCAATCTGGCATGAAG |
| *Cxcl1* | GGCGCCTATCGCCAATG | CTGGATGTTCTTGAGGTGAATCC |
| *PAI-1* | ACAGCCTTTGTCATCTCAGCC | CCGAACCACAAAGAGAAAGGA |
| *Occludin* | ATTCCATCAGTTTCCTATCT | ACCAGGACCTTTCTTGAC |
| *ZO-1* | ACAGGCCATTACGAGCCTCT | GGAGGCTGTGGTTTGGTAGC |
| *Cldn2* | TCTCAGCCCTGTTTTCTTTGG | GGCGAGCAGGAAAAGCAA |
| *Cldn3* | TCATCACGGCGCAGATCA | CTCTGCACCACGCAGTTCA |
| *Cldn15* | GGCGGCATCTGTGTCTTCTC | TGGTGGCTGGTTCCTCCTT |
| *Jam3* | CACTACAGCTGGTACCGCAATG | CTGGGATTGGCTCTGGAATC |

**
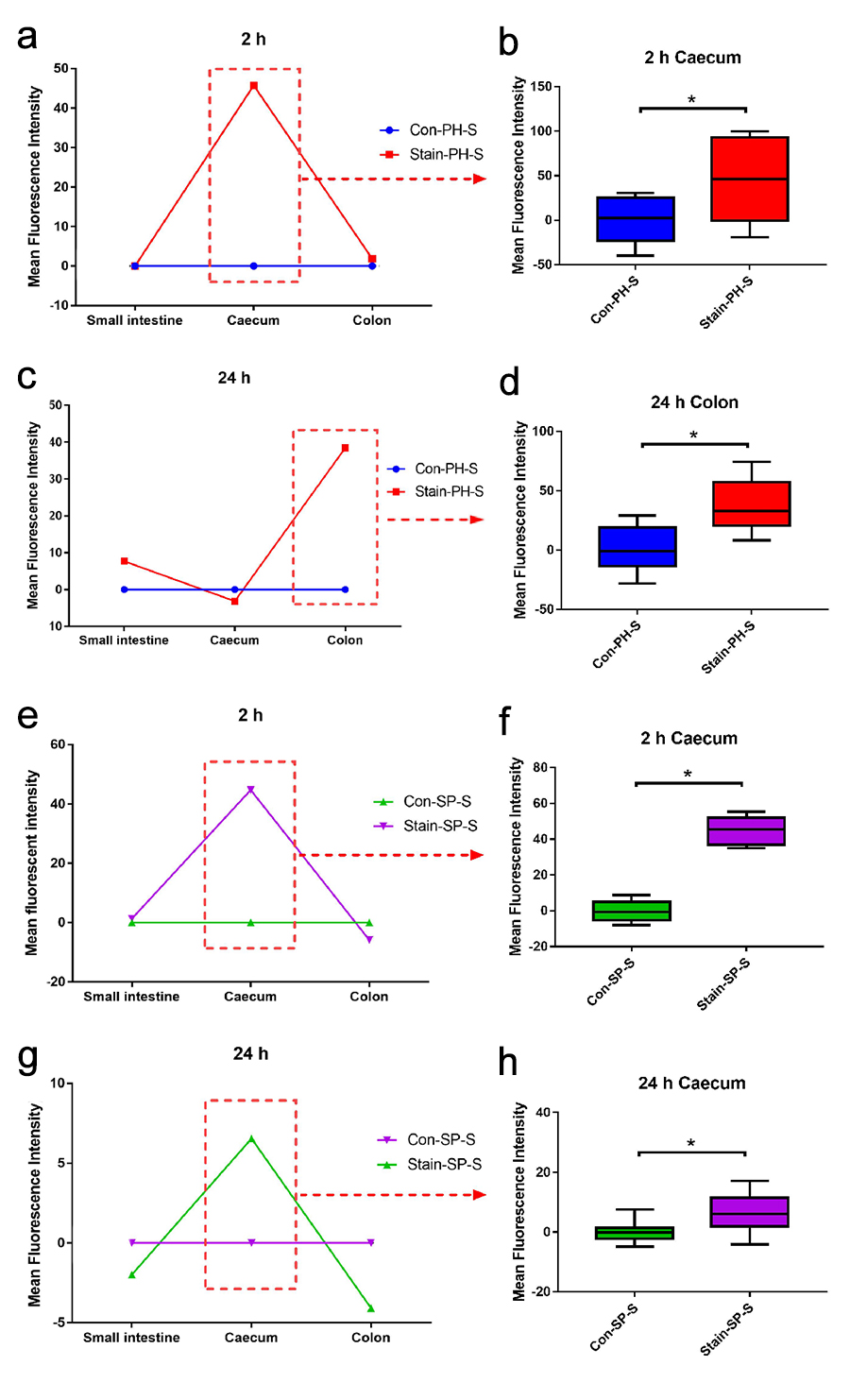
**

**Appendix Figure 1. The periodontitis salivary microbiota could persist in the intestinal tract for at least 24 hours (n = 6 in each group).** (a-h) The distribution of fluorescence-positive bacteria in the gastrointestinal tract (small intestine, caecum, and colon) at 2 hours and 24 hours after gavage. Data are compared to the Con-PH-S group (a-d) or Con-SP-S group (e-h). Statistics are expressed as the mean or mean ± SD. Statistical significance was determined using a t-test. **P* < 0.05.

**
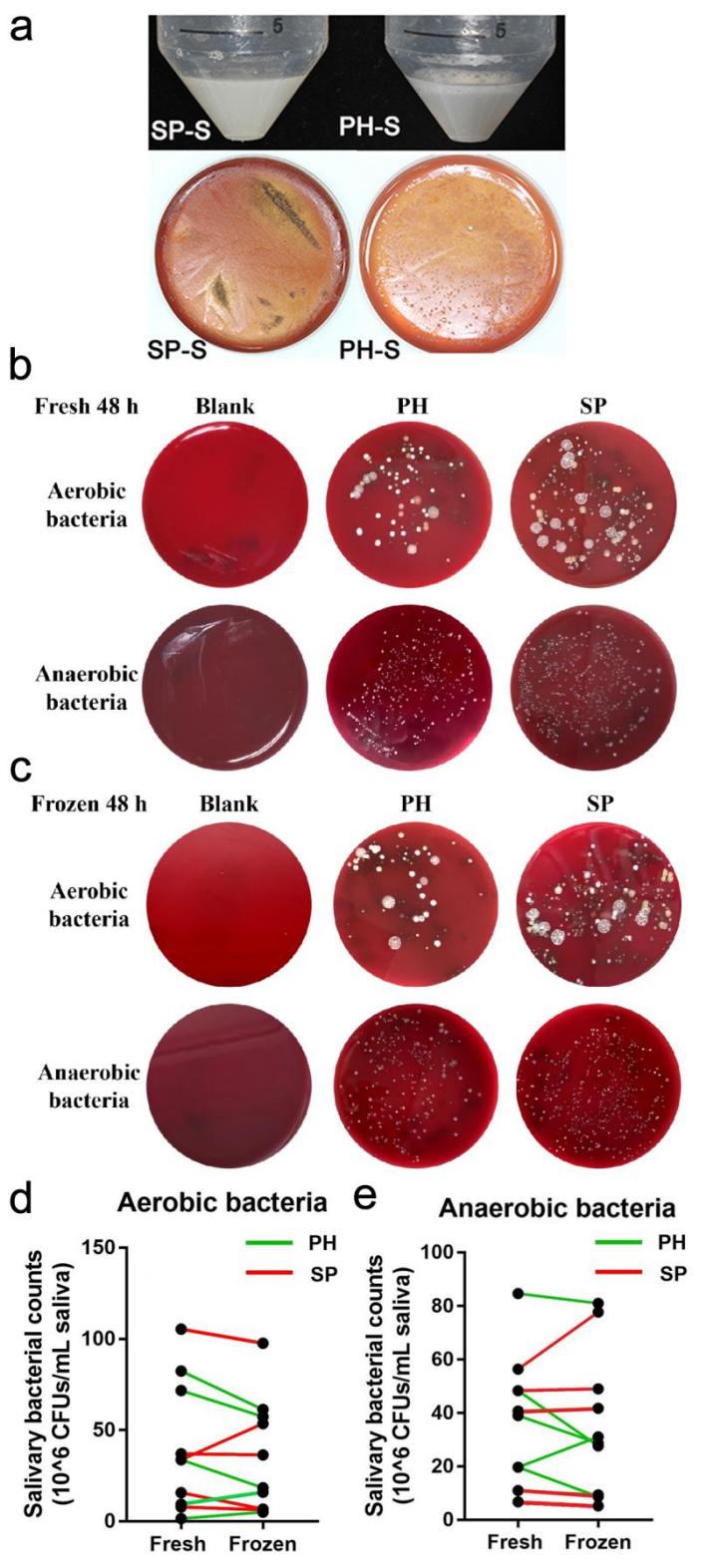
**

**Appendix Figure 2. Salivary bacteria were still alive after freezing.** (a) Representative images of the appearance of the saliva and bacterial colonies of thawed and pooled saliva anaerobically cultured on BHI agar for 14 days. (b-e) Salivary bacterial counts of the PH group (green line, n = 5) and SP (red line, n = 5) group before and after freezing under anaerobic and aerobic conditions for 48 h (dilution ratio 1:1000). Data are expressed as the mean. Statistical significance was determined using a t-test.

**Assessment of Salivary Bacterial counts**

To confirm the good preservation of bacteria, salivary bacteria were cultured after cryopreservation. One hundred microlitres of thawed and pooled saliva samples were placed into brain heart infusion (BHI) broth medium made by 3.7 g/100 mL brain heart infusion (Remel Inc, Lenexa, USA), 0.5 g/100 mL yeast extract (Remel Inc, Lenexa, USA), 1 mg/L hemin (Alfa Aesar, Haverhill, USA), 1 mg/L menadione (Alfa Aesar, Haverhill, USA), 1.8 g/100 mL agar (Biosharp, Anhui, China), and 5 mL/100 mL defibrinated sheep blood (Guangzhou Ruite Biotechnology Co. Ltd, Guangzhou, China) and cultured at 37°C under anaerobic conditions for 14 days.

The collected saliva samples from each individual were divided into fresh and frozen portions. The frozen portions were mixed with an equal volume (w/v) of phosphate-buffered saline (PBS) containing 20% glycerol and stored at −80°C for 7 days. Then, the frozen saliva samples were thawed. A total of 1 mL of fresh saliva and 2 mL of thawed saliva (containing 1 mL of saliva and 1 mL of glycerol/PBS) from each participant were centrifuged at 1,000 × *g* for 5 min to remove food residues and exfoliated cells and then centrifuged at 3,300 × *g* for 10 min at 4°C to collect the microbiota. Then, the salivary microbiota was suspended in 1 mL of PBS. The salivary microbiota suspensions were serially diluted to 1 in 1000 and10,000 in PBS and used to inoculate lysed blood agar plates. One series of plates were incubated under anaerobic conditions (10% CO_2_, 10% H_2_, 80% N_2_) at 37°C for 48 h, and the other in air at 37°C for 48 h. After the incubation period, the resulting colonies were counted to determine the number of colony forming units (CFUs) per 1 mL.
